# A Longitudinal Study Regarding the Health Profile of the 2017 South African Hajj Pilgrims

**DOI:** 10.3390/ijerph18073607

**Published:** 2021-03-31

**Authors:** Abdulaziz Mushi, Yara Yassin, Anas Khan, Badriah Alotaibi, Salim Parker, Ozayr Mahomed, Saber Yezli

**Affiliations:** 1 The Global Centre for Mass Gatherings Medicine, Ministry of Health, Riyadh 12341, Saudi Arabia; yyassin@moh.gov.sa (Y.Y.); khanaa@moh.gov.sa (A.K.); bmalotaibi@moh.gov.sa (B.A.); SYezli@moh.gov.sa (S.Y.); 2 Department of Emergency Medicine, College of Medicine, King Saud University, Riyadh 12372, Saudi Arabia; 3 Division of Infectious Diseases and HIV Medicine, Department of Medicine, University of Cape Town, Groote Schuur Hospital, Cape Town 7925, South Africa; salimparker@yahoo.com; 4 Department of Public Health Medicine, University of KwaZulu Natal, Durban 4051, South Africa; ozayr411@gmail.com

**Keywords:** South Africa, Hajj, pilgrimage, healthcare, public health, respiratory tract infection

## Abstract

The Hajj mass gathering annually attracts over two million Muslim pilgrims worldwide to the Kingdom of Saudi Arabia (KSA). We aimed to establish demographics and health profiles for the South African pilgrims performing the 2017 Hajj. **Methods:** This is a longitudinal survey-based study conducted on 1138 adult South African pilgrims in two phases (during and post-Hajj). Data on demographics, vaccination status, underlying health conditions, pre-Hajj training, health promotion, travel history, and health issues during and post-Hajj were collected using pre-designed questionnaires. **Results:** Participants had a mean age of 49.2 years (SD = 13.3; range 18–81), with a male: female ratio of 1.2:1. The majority of pilgrims were married (88.2%), of Indian/Asian background (73%), and literate (>99%). Nearly all pilgrims were vaccinated against meningococcal disease and yellow fever, but only 23.7% were vaccinated against Influenza. Hypertension, diabetes, and elevated cholesterol levels were the most common underlying health conditions reported by 22.6%, 13.2%, and 11.5% of pilgrims, respectively. One month after return to South Africa, nearly 65% of pilgrims reported illness during Hajj, while 40% reported falling ill post event upon return to South Africa. Nevertheless, only a few were admitted to hospitals (12 during Hajj and 15 post-Hajj). Among ill pilgrims, respiratory symptoms were the most commonly experienced symptoms during (70.2%) and post-Hajj (82.2%). Other symptoms such as walking-related symptoms include symptoms directly related or mainly caused by walking (e.g., leg pain, sore feet, blisters on the feet), dehydration, and gastrointestinal tract symptoms reported during Hajj. Medication to treat respiratory symptoms and antibiotics were the most commonly used medications during and post-Hajj. Having an underlying health condition was an independent predictor of falling ill during or post Hajj. **Conclusion:** Our study indicates that a sizable proportion of South African pilgrims are elderly with underlying health conditions and most contract respiratory tract infections during and post Hajj. Our study highlights the need for systematic collection of prospective pilgrims’ demographics and health data and more attention to post-Hajj health follow-ups of pilgrims.

## 1. Introduction

Hajj is an annual religious mass gathering in Makkah, Saudi Arabia (KSA) and attracts over two million Muslim pilgrims. Although the actual Hajj rite does not last for more than five days, many pilgrims extend their stay in Makkah and the surrounding cities, usually arriving early, departing late, or both for various reasons. Pilgrims originate from over 180 different countries with varying ethnicities, cultures, languages, and socio-economic and health backgrounds [1]. A large proportion of Hajj pilgrims are elderly, many with underlying health conditions and on medications. In addition, Hajj involves demanding religious rites in crowded settings and often in the outside hot environment. As such, the pilgrimage is associated with several health risks include communicable and worsening or complications of non-communicable disease, injuries and heat-related illnesses [2]. These health risks do not always originate in the Kingdom, and their effects are often not confined to the Hajj period but are sometimes felt long after Hajj is completed [3,4].

Mass gatherings such as the Hajj pose significant public health challenges and stress the host’s health system. Therefore, timely and appropriate planning for the event is crucial. KSA authorities employ a well-established system for planning, communication, public health, and Hajj safety issues [5]. This includes delivering health services during the event, free for pilgrims through numerous hospitals, primary healthcare centers, and other health facilities [6]. Besides, some countries operate medical units during the Hajj season to care for their pilgrims, minimizing language or cultural barriers to healthcare. There is an increasing number of publications on pilgrims’ health conditions during Hajj, including data on morbidity and mortality, generally specific to certain countries [7,8,9]. However, data on demographics and health profile of prospective pilgrims are not systematically collected and shared with Saudi Health Authorities pre-Hajj, nor is data on pilgrims’ health post event [10]. Such information would greatly aid the public health planning for Hajj, including risk assessment and the development of appropriate health promotion messages. Additionally, such information would be useful to determine health service needs for the pilgrimage, the impact of Hajj on the health of pilgrims post event and preserve global health security in case of Hajj-linked outbreaks of diseases post event [10]. Data on prospective pilgrims’ demographics and health profile is particularly useful for countries with a Hajj pilgrim population that is significantly different in its profile from that of the country’s general population. This includes countries like South Africa, where Muslims represent only 1.5–2% of the total population, most of whom are of Indian/Asian descent with distinct socio-economic characteristics to that of the general population [11]. Around 2500 Muslim South Africans gather to perform Hajj every year [4]. This highlights the great importance of creating a Hajj health profile for this cohort.

We aimed to collect data on South African pilgrims and their health pre, during, and post Hajj to determine their demographics and health profiles and the impact of Hajj on their wellbeing.

## 2. Methodology

### 2.1. Study Design and Data Collection

This longitudinal survey-based study was conducted in two phases, through which data was collected from pilgrims who agreed to participate, using pre-designed questionnaires. The questionnaire was in English administered through trained investigators. The questionnaire was developed reviewing available publications in the literature but was tailored for the study objectives and Hajj setting [12,13,14,15,16,17,18]. The content of the questionnaire was then reviewed by two public health experts with Hajj experience. For the two phases, they were as follows:

Hajj phase: Data on pilgrims’ demographics (e.g., age, gender, country of origin, level of education), vaccination status, underlying health conditions, pre-Hajj training, and health promotion, as well as travel history, was collected during Hajj in KSA from the 23rd of August to the 20th of September 2017.

Post-Hajj phase: Pilgrims were followed-up, one month after their return to South Africa from Hajj, through phone call interviews. Data was collected on health issues during and post-Hajj in South Africa from the 13th of October 2017 to the 5th of February 2018. This included information on illness symptoms, symptoms, diagnosis, medications prescribed and visits to healthcare facilities or hospitals’ admissions.

### 2.2. Study Population

Our study targeted all adult South African pilgrims (>18 years old) who attended the 2017 Hajj and consented to participate. A total of 1138 pilgrims were enrolled in this study.

### 2.3. Statistical Analysis

The study population’s characteristics were summarized as frequencies and percentages for categorical variables and means, range, and standard deviations (SDs) for quantitative variables. The association between explanatory variables and outcomes was evaluated by the Chi-Square test or Fisher’s exact test, as appropriate [19]. All significance tests were two-sided, and a *p*-value of <0.05 was considered statistically significant. Odds ratios with 95% CIs were computed to assess the presence and degree of association between the dependent versus independent variables. Variables with *p*-value <0.05 at the bi-variate analysis were taken for multivariate analysis. All analyses were performed using IBM SPSS Statistics version 25.0 (IBM Corp., Armonk, NY, USA).

### 2.4. Ethics and Confidentiality

All study participants were briefed about the study and gave verbal consent before enrolment. The study was approved by the King Fahad Medical City Ethics Committee, the Institutional Review Board (IRB log: 17-244E), and the Biomedical Research Ethics in the University of KwaZulu-Natal, South Africa (IRB log: BE491/17).

## 3. Results

### 3.1. Characteristics of the Study Population

#### 3.1.1. Demographics

**Figure 1:**
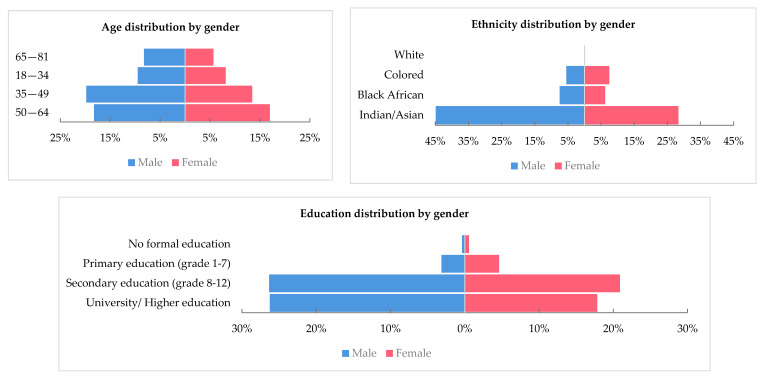
Age, ethnicity and level of education distribution by gender among the study population.

Of the 2500 South African pilgrims who attended the 2017 Hajj, 1138 (45.5%) were eligible and agreed to participate in the study; among participants, 80.5% was the response rate for post hajj follow up. The participants had a mean age of 49.2 years (SD = 13.3; Range 18–81), with a male:female ratio of 1.2:1. Most pilgrims, 91% (1000/1098), had at least a secondary education, and nearly all could read 99.7% (1124/1127) and write 99.6% (1108/1112). The age and level of education distribution by gender are presented in Figure 1.

A large proportion of pilgrims, 73% (476/652), were of Indian/Asian background (Figure 1), and the majority (>97%) were South African nationals, residing in South Africa, 15% (167/1112) of respondents reported traveling outside of their country of residence in the past six months, mainly visiting Saudi Arabia and the United Arab Emirates (UAE). Nearly half of the pilgrims reported performing Umrah, but only 10% (113/1126) had previously performed Hajj, mostly more than five years ago. Around 40% (413/1011) of pilgrims stated that they have travel insurance, including Hajj travel (Table 1).

#### 3.1.2. Vaccination and Underlying Health Conditions

**Figure 2:**
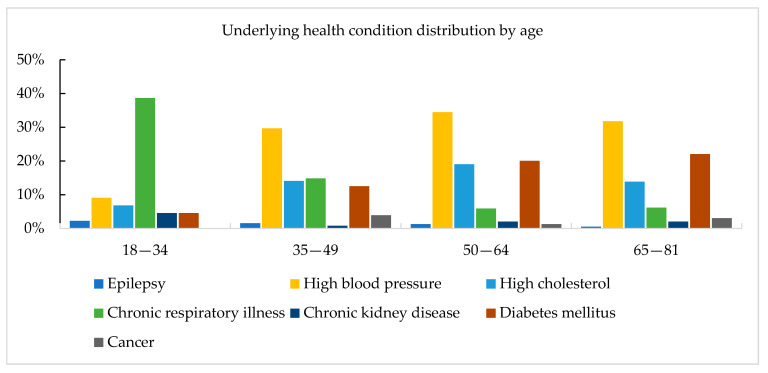

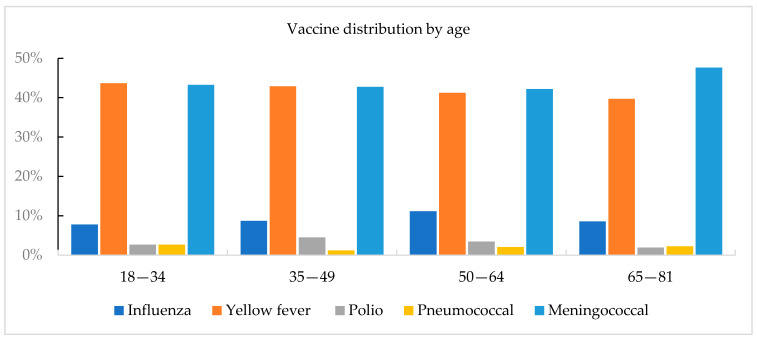
Vaccinations and underlying health conditions distrubtion by age groups among the study population.

The majority of pilgrims 98.4% (1113/1131) reported being vaccinated against meningococcal disease using the quadrivalent conjugate vaccine. About 95.2% (1082/1137) were vaccinated against yellow fever, 23.7% (241/1019) received the influenza vaccination, and only 4.7% (48/1019) were vaccinated against pneumococcal disease. Hypertension, diabetes (mainly Type 2), and elevated cholesterol levels were the most common health conditions reported by 22.6% (239/1057), 13.2% (139/1057) and 11.5% (122/1057) of pilgrims, respectively. Allergy was reported in only 8% (84/1058) of pilgrims (mainly to medications and environmental factors), while chronic respiratory illnesses were reported in 6.8%. (72/1055) (Table 2). The distributions of vaccinations and underlying health conditions by age groups are presented in Figure 2. For most underlying health conditions, the prevalence was higher among the older age groups.

#### 3.1.3. Pre-Hajj Training and Health Promotion

Regarding training and health promotion before arrival to KSA, 90.5% (1019/1126) of pilgrims declared that they had received training on the Hajj journey and rituals. Similarly, most pilgrims reported that they knew where to seek medical care in KSA 76.6% (863/1126) and the location of their medical missions during Hajj 66.3% (738/1113). About 66.5% (733/1103) of pilgrims reported having been briefed about possible health risks to expect during their stay in KSA, mainly related to heat and respiratory illnesses (Table 3). Most of the specific health messages received by pilgrims were regarding hand hygiene 44.7% (505/1131), heat-related diseases 42.4% (479/1131), and cough etiquette 33.6% (380/1131). Most pilgrims reported that the health messages they received were clear and easy to understand (Table 3).

#### 3.1.4. Health Profile upon Arrival to Saudi Arabia

Over 25% (273/1086) of the participants mentioned suffering from a cough upon arrival to Saudi Arabia, 9% (89/1083) mentioned having a fever (acute fever in the vast majority of cases; <2 weeks), and 9.7% (105/1083) reported having diarrhea. Vomiting appeared in only 2.3% (25/1056) of participants and lasted for less than three days in most cases, while 11% (119/1088) reported suffering from a headache, mostly for less than a week.

### 3.2. Health Profile during and Post-Hajj

About 65.1% (596/916) of the participants had fallen ill during Hajj, and 31.6% reported suffering at least two illness episodes during the pilgrimage. The mean duration of illness episodes during Hajj was 3.5 days (SD = 2.9). Nearly 40% (365/915) of pilgrims also reported that they got sick post-Hajj, displaying symptoms on average four days (SD = 5.6) after returning home. There were significantly more pilgrims who fell ill during Hajj than post Hajj (*p*-value = 0.00). Only 12 participants were admitted to the hospital during Hajj, most 66.6% for only one day. Post-Hajj, 15 pilgrims were admitted to hospitals in South Africa for a mean duration of 6.4 days (SD = 5.7).

#### 3.2.1. Experienced Symptoms

Respiratory symptoms were the most commonly experienced symptoms among the pilgrims during and post-Hajj (*p*-value = 0.073). Among ill pilgrims, respiratory symptoms were found to be significantly higher post-Hajj than during Hajj, with 70.2% (413/588) and 82.2% (287/349), respectively (*p*-value = 0.000). On the other hand, gastrointestinal tract (GIT) symptoms were significantly more common during Hajj compared to post Hajj (5.4% (32/588) vs. 1.4% (5/349), *p*-value = 0.002). Similarly, dehydration was only reported during Hajj in 10.9% of ill pilgrims. Walking-related symptoms (these include symptoms that are directly related or main caused by walking, e.g., leg pain, sore feet, blisters on the feet) and injuries were reported in 10% (59/588) of ill participants during Hajj and in 7.1% (25/349) post-Hajj (*p*-value = 0.137).

In the bivariate analysis, having an underlying health condition (OR = 1.61; 95% CI = 1.18–2.19; *p* = 0.003) and not having previously performed Hajj (OR = 1.57; 95% CI = 1.00–2.47; *p* = 0.047) were significantly associated with increased risk of falling ill during Hajj. In the multivariate analysis, pilgrims with underlying health conditions were 1.6 times more likely to fall ill during Hajj (OR = 1.60; 95% CI = 1.17–2.19; *p* = 0.003). Similarly, pilgrims with underlying health conditions were 1.4 times more likely to fall ill post-Hajj (OR = 1.41; 95% CI = 1.08–1.85; *p* = 0.011).

#### 3.2.2. Diagnosis and Prescribed Medications

Regarding medical diagnosis, our study results showed that diagnosis with respiratory and hypertension as well as muscle-related illness were all significantly more common in Hajj than post Hajj (Table 4). Respiratory illnesses were by far the most common diagnosis among pilgrims both during 32.1% (168/523) and post Hajj 44.4% (119/268). Similarly, the medication used to treat respiratory and gastrointestinal symptoms, analgesic medication, and vitamins were all significantly more commonly prescribed during Hajj than post Hajj (Table 4). In contrast, there was no significant difference in prescribing of antibiotics between the two periods (66.2% (346/523) during Hajj vs. 68.7% (184/268) post Hajj, *p*-value = 0.479).

#### 3.2.3. Treatment Source

The South African Medical Mission Clinic was the most visited healthcare provider during Hajj (87.7% (522/596)), while private hospitals were the most visited post-Hajj in South Africa 57% (208/366). Furthermore, self-treatment was significantly higher post-Hajj compared to during Hajj (37.5% (137/569) vs. 17.1 (102/366) *p*-value = 0.000).

## 4. Discussion

Our study aimed to collect data on demographics and the health of South African pilgrims pre, during and post Hajj. The study enrolled all adult pilgrims who agreed to participate, encompassing nearly half of all the South African pilgrims who participated in the 2017 Hajj. Hence, giving the best representation of the demographics and health profile of South African pilgrims to date. The study also reports on health issues experienced by pilgrims during and post Hajj and highlights the high rates of respiratory tract infection and antibiotic use during and post pilgrimage.

We report that South African pilgrims represent the general Hajj population in relation to gender, age, and underlying health conditions. The General Hajj population has a slightly higher proportion of males, is dominated by older pilgrims, many with underlying health conditions [4,20,21,22]. In our study, 55.8% of pilgrims were males, which is similar to the proportion of males in the overall Hajj population of 2017 (56.7%) [21]. Similarly, South African pilgrims had a mean age of 49.2 years, and 49.4% were aged 50 years or older. While some studies on pilgrims from specific countries reported higher mean age for pilgrims (57–66 years) [8,23,24], our results are in accordance with data on the overall Hajj population where over 50% are aged 50 years or older [20]. Yet, it appears that the proportion of South African pilgrims aged 50 years or older had increased over the years from 34.5% in 2006 to 40% in 2007 to 49.4% in the current study [25].

Additionally, our study revealed a very high literacy level among South African pilgrims, with a large proportion (91.1%) having a secondary of higher education. Reported levels of education among pilgrims varied depending on the studied populations. One study among pilgrims from South Africa, Nigeria, Pakistan, Egypt, Iraq, and Indonesia in 2017 found that 83.7% had secondary or higher education [26]. A similarly high proportion was reported among Indonesian and Australian pilgrims [27,28]. On the other hand, a study among pilgrims from South Africa, Nigeria, Pakistan, Afghanistan, and Bangladesh in 2015 reported that only 34.2% had secondary or higher education [4]. However, South African pilgrims’ level of education is higher than that of the general South African population, wherein 2016, only 34.6% of adults 25 years or older have at least a secondary education [11]. This discrepancy is probably related to the fact that most South African pilgrims, as indicated by this study’s results, are of Indian/Asian descent, with a different socio-economic profile compared to most of the South African population [11,29].

In this study, 40% of respondents had at least one underlying health condition, with 22.6% having hypertension and 13.2% being diabetic. These results are in accordance with other reports showing that a sizable proportion of Hajj pilgrims have underlying health conditions, with diabetes and hypertension being the most common [30]. Studies among pilgrims from various countries found that 27–58% have at least one underlying health condition [4,20,21,22]. The prevalence of hypertension among Hajj pilgrims ranged between 10–47%, while <10–33% were reported to be diabetic [23,24,31,32,33]. Our results are within the above reported ranges and close to those for the South African general population. The World Health Organization (WHO) estimates that 26.9% of adult South Africans in 2015 had hypertension, and in 2016, the prevalence of diabetes in the country was 9.8% [34]. Data on underlying health conditions among prospective pilgrims would be of great value to ensure that pilgrims receive targeted health education messages on managing their conditions during Hajj and plan for the appropriate health services delivery. This is particularly important given that pilgrims with underlying health conditions are over-represented among hospital and Intensive care unit (ICU) admissions as well as deaths during the pilgrimage [8,9,14,15,35].

Given the potential for the rapid spread of infectious diseases during Hajj, the Hajj health requirements for pilgrims are focused on vaccination to minimize the risk of disease and outbreaks during and post event. Meningococcal vaccination has been compulsory for all pilgrims, including South Africans since 1988 [36]. Besides, the Saudi Ministry of Health highly recommends that all pilgrims receive the influenza vaccination, though it is not compulsory [37]. The results of our study are in line with others who reported high, but not always full compliance, with meningococcal vaccination and poor uptake of the non-recommended vaccination. For instance, in our study, 98.4% of South African pilgrims reported being vaccinated against meningococcal disease, which is similarly high to what has been found in other recent studies [36]. Uptake of Influenza and pneumococcal vaccines in our study was low, especially in relation to the latter. Similar results were reports among multinational cohorts of Hajj pilgrims [38]. Interestingly, although vaccination against yellow fever is not compulsory for South African pilgrims [37], over 95% of pilgrims reported being vaccinated. This may be due to the fact that many South Africans often fly to Saudi Arabia via Ethiopia and Kenya, where the yellow fever vaccination is mandatory for all arrivals [25].

We investigated the impact of Hajj on pilgrims’ health through tracing developed symptoms, visits to healthcare facilities, medical diagnosis, and dispensing of medications during and after the event. Our study results confirm previous reports that respiratory infections are the most common diseases experienced by pilgrims and are among the main reason for visits to healthcare facilities and dispensing of medications during Hajj, including among South African pilgrims [9,12,24,25]. Besides, we report that respiratory symptoms were more common post-Hajj than during Hajj. Several studies found the significant acquisition of respiratory pathogens and the development of respiratory symptoms among pilgrims post-Hajj [12,39]. Mixing of pilgrims in crowded conditions during Hajj facilitates transmission of respiratory pathogens and development of respiratory symptoms during Hajj. The prolonged incubation period of some respiratory diseases may also explain the prevalence of respiratory illness post pilgrimage [40]. Similar to other reports, we found that the gastrointestinal tract (GIT) symptoms and illness were much less common among pilgrims than respiratory infections [9,41]. Additionally, these were mainly seen during Hajj, which may also be linked to the relatively short incubation period of the GIT disease-causing organisms [42]. In addition to infections, symptoms associated with walking were also reported during Hajj. This is not surprising given that pilgrims walk for an estimated distance of 58 km during Hajj’s five days to perform its rituals [18].

The pattern of medication prescribed to pilgrims pre-and post-Hajj was in accordance with symptoms experienced by pilgrims. Given infections (especially respiratory tract infections) were the most common illnesses among pilgrims, medications to relieve symptoms of such infections and well as antibiotics were commonly prescribed. Besides, vitamins likely used to boost the pilgrims’ nutrients and immune system, as well as analgesic medications were also commonly prescribed. A recent study on medication prescribing patterns among outpatients in Hajj found that the most common classes of prescribed drugs were anti-inflammatory medicines, analgesics and antipyretics, antibacterials, cough, and cold preparations, antihistamines, and drugs for acid related disorders [43]. Similarly, data from the Indian medical mission of the 2016 Hajj revealed that analgesics, antibacterials, antacids, antihistamines and diabetes and hypertension medications were the most common drugs prescribed to pilgrims [9,43]. Interestingly, antibiotics were commonly prescribed both during and post-Hajj. Use of antibiotics is common during Hajj and may have an impact post-pilgrimage. For example, Hoang et al. reported that 47.6% of pilgrims used antibiotics during Hajj, and that antibiotic intake was associated with increased carriage of certain respiratory pathogens and increased frequency of persistent symptoms post-Hajj [44,45].

Hajj is a unique experience and pilgrims need to be adequately informed about all aspects of the pilgrimage prior to arrival to KSA, including health risk and how to access medical services during the event. In our study, while a sizable proportion of pilgrims had visited KSA and performed Umrah previously, for most of them, it was their first time performing Hajj. While these two mass gatherings take place in the same location, they different in many aspects including crowdedness, duration, rituals, and health risks [1], which highlights the importance of providing pilgrims with training programs and information that are specific to Hajj. We found that most South African pilgrims had some form of training about Hajj prior to arrival to KSA, including health risks and how and where to seek medical assistance during the event. Most of the health information given to pilgrims was about respiratory tract infection and health-related illness and how to prevent them. This is not surprising given that respiratory illness is the leading cause of morbidity and hospitalization during Hajj and significantly contributes to mortality at the event [1,7,9,14,16].

Additionally, given the Hajj location and a heat-vulnerable pilgrim population, heat-related illnesses are considered public health threats during the event, especially when it is held in the summer months, as was the case during our study period [46]. However, we note a lack of health information for pilgrims regarding non-communicable diseases and management of underlying health conditions such as diabetes and hypertension, which may be associated with a lack of emphasis on these conditions in the Saudi Hajj health regulations for pilgrims [17]. Non-communicable diseases, especially cardiovascular diseases, are the leading cause of mortality during Hajj [16], and health information on how to prevent and manage conditions that could lead to cardiovascular events during Hajj should be disseminated among pilgrims Pre-Hajj. While pilgrims also receive health promotion messages and information on preventive measures and health services while in KSA, health authorities in the countries of origin are encouraged to provide health education for pilgrims prior to arrival to KSA for Hajj. This is because pre-Hajj health advice is an essential factor contributing to increased compliance with pilgrims’ recommended health measures [47,48].

Given the international nature of the Hajj, the crowded conditions at the event and the constant movement of pilgrims during the Hajj journey, tracking pilgrims’ health and provision of health services can be challenging. Active and real-time surveillance are applied during Hajj, including a recently implemented syndromic and event-based health early warning system [49,50]. However, post-Hajj surveillance, particularly at pilgrims’ countries of origin is still inadequate. The implementation of a unified surveillance system that collects data on pilgrims’ health pre-, during and post-Hajj, would fill this gap [10]. Utilizing new technologies for evaluating and monitoring pilgrims’ health at distance and in real-time, as well as providing consultation and medical assistance remotely, can be useful in such a crowded event. Various technologies to monitor patients’ vital signs and other health parameters at distance have been developed [51,52]. These, in combination with telemedicine, could prove beneficial in Hajj, including during the current COVID-19 pandemic [53]. It is worth nothing that demographics and health profiles of prospective pilgrims, as well as risk communication messages, can vary significantly depending on public health policies introduced due to specific health risks. This includes times when Hajj takes place amid global pandemics or public health emergency of international concern. For example, due to the COVID-19 pandemic, the 2020 Hajj was a scaled down Hajj with only a 1000 (mainly younger and healthy) pilgrims residing in KSA [54].

This study has some limitations. Although we systematically invited all eligible pilgrims to participate, only 45.5% of pilgrims agreed and were enrolled. Additionally, we used questionnaires and interviews to collect information from pilgrims during and post Hajj. As such, responses obtained are prone to information bias, particularly recall bias. Nevertheless, our study is the first to collect information on the health of nearly half of the South African pilgrim population pre- during and post Hajj and reporting on Hajj’s impact on the health of these pilgrims. Future studies should investigate a more significant number of pilgrims from more countries with larger pilgrim populations, such as India and Indonesia.

## 5. Conclusions

In summary, our study indicates that the South African Hajj population is representative of the general Hajj population in terms of gender composition and age and that of the South African (in particular the Indian/Asian) population in terms of socio-economic status and underlying health conditions. Pilgrims appear to have been informed about where to seek medical assistance during Hajj and about some of the leading health risks during Hajj. Additionally, pilgrims did adhere to the required vaccinations for the pilgrimage. Around two-thirds of pilgrims had fallen ill during Hajj and one-third reported suffering illness post event. In both instances, respiratory infections were by far the most common cause of illness. A pattern of medication prescribing both during and post Hajj was in accordance with illnesses and symptoms reported by pilgrims, with frequent use of antibiotics and medications to relieve symptoms of respiratory illnesses. Our results highlight the need for a unified data sharing platform for Hajj pilgrims among Hajj stakeholders that capture pilgrims’ demographics and health information pre, during and post event [10]. As such, supporting public health preparedness and planning for the event and improving response capabilities and health services delivery during and post the mass gathering. Until such a platform is developed, results of studies like ours can be used to assist in the above endeavors.

## Figures and Tables

**Table 1:** Demographic characteristics.

Variable	Number (n)	Percentage (%)
**Pilgrims enrolled**	1138 *	
**Gender**	**1114**	
Male	622	55.8
**Age Group**	**1130**	
18–34	199	17.6
35–49	372	32.9
50–64	398	35.2
65–81	161	14.2
**Highest education level**	**1098**	
No formal education	10	0.9
Primary education (grade 1–7)	88	8.0
Secondary education (grade 8–12)	519	47.3
University/Higher education	481	43.8
**Read**	**1127**	
Yes	1124	99.73
**Write**	**1112**	
Yes	1108	99.64
**Marital status**	**1125**	
Married	992	88.2
Single	88	7.8
Others	45	4.0
**Ethnicity**	**652**	
Indian/Asian	476	73.0
Black African	92	14.1
Colored	83	12.7
White	1	0.2
**Travel insurance**	**1011**	
No	446	44.1
Yes, including Hajj	348	34.4
Yes, without Hajj	65	6.4
Do not know	152	15.0
**Previously performed Hajj**	**1126**	
Yes	113	10.0
** Hajj performance, how long ago **	** 484 **	
Last year	3	6.3
1–5 Years	4	8.3
More than 5 years	41	85.4
**Previously performed Umrah**	**1114**	
Yes	495	44.4
** Umrah performance, how long ago **	** 224 **	
Less than a Year	16	7.1
1–5 Years	137	61.2
More than 5 years	71	31.7
**Travel outside South Africa in the past 6 months**	**1112**	
Yes	167	15.0
**Country frequency (Most visited countries in the past 6 months)**	**225**	
India	18	8.0
Mauritius	10	4.4
Saudi Arabia	23	10.2
UAE **	30	13.3
Other (# of countries less than 4%)	144	64.1
**Currently pregnant (pregnancy status)**	**475**	
No	460	96.8
Yes	7	1.5
Do not know	8	1.7
**Pregnancy months**	7	
First Trimester	6	85.8
Second Trimester	1	14.3

* Bold numbers; represent the total number in each category.** UAE; United Arab Emirates.

**Table 2:** Vaccination and underlying health conditions among enrolled participants.

Variable	*n*	Yes	%
**Vaccination**			
Yellow fever vaccination	1137	1082	95.2
Polio vaccination	1018	89	8.7
Influenza vaccination	1019	241	23.7
Pneumococcal vaccination	1019	48	4.7
Meningococcal vaccination	1131	1113	98.4
**Health conditions**			
**High blood pressure**	1057	239	22.6
**Diabetes mellitus**	1057	139	13.2
Type 1	67	8	11.9
Type 2	67	59	88.1
**Heart attack**	1055	32	3.0
**Chronic respiratory illness**	1055	72	6.8
**Chronic kidney disease**	1056	15	1.4
Hemodialysis	15	5	33.0
**Epilepsy**	1055	9	0.9
**Angina**	1055	30	2.8
**High cholesterol**	1057	122	11.5
**Cancer**	1055	16	1.5
**Allergy**	1058	84	7.9
Environment	90	53	58.8
Food	90	8	8.8
Medication	90	35	38.8
Other	90	2	2.2
**Other condition**	1054	17	1.6

**Table 3:** Training and health promotion before arrival to KSA *.

Variable	*n*	Yes	%
**know where to seek medical care in KSA before arrival**	1126	863	76.6
**know where medical mission clinics location in KSA before arrival**	1113	738	66.3
**Hajj training received before arrival to KSA**	1126	1019	90.5
**Information about health risks in KSA before arrival**	1103	733	66.5
Heat related illnesses	127	46	36.2
Respiratory related illness	127	52	40.9
Gastrointestinal disorders	127	15	11.8
Other	127	77	60.6
**Information on how to seek medical help received before arrival to KSA**	1113	976	87.7
**Health messages received before arrival to KSA**	1106	625	56.5
Hand hygiene	1131	505	44.7
Heat-related illnesses	1131	479	42.4
Cough etiquette	1131	380	33.6
No messages received	1131	215	19.0
Others health messages received	1131	36	3.2
**Health messages received before arrival were easy to understand**	602		
No	602	29	4.8
Yes	602	573	95.2

* KSA; kingdom of Saudi Arabia.

**Table 4:** Health issues during and post-Hajj.

Variable	During			Post			*p* Value
	n	Yes	%	n	Yes	%	
**Fall ill**	916	596	65.1	915	365	39.9	0.000
**Symptoms**							
Respiratory symptoms	588	413	70.2	349	287	82.2	0.000
GIT * symptoms	588	32	5.4	349	5	1.4	0.002
Fever	588	68	11.6	349	34	9.7	0.387
Dehydration	588	64	10.9	349	0	0.0	0.000
Walking Related symptoms	588	59	10.0	349	25	7.1	0.137
Other symptoms	588	226	38.4	349	95	27.7	0.000
**Diagnosis**							
Respiratory disease	536	375	70.0	309	252	81.5	0.000
Muscular illnesses	536	28	5.2	309	1	0.3	0.000
Blood pressure disorders	536	17	3.2	309	1	0.3	0.006
Gastrointestinal disorders	536	15	2.8	309	3	1.0	0.076
Urinary tract disease	536	8	1.5	309	1	0.3	0.102
Diabetes	536	8	1.5	309	0	0.0	0.260
Ear infection	536	4	0.7	309	2	0.6	0.087
Allergy	536	4	0.7	309	0	0.0	0.161
Cancer diagnosis	536	1	0.2	309	0	0.0	0.634
Heat related illnesses	536	1	0.2	309	0	0.0	0.634
**Medications**							
Antibiotic	523	346	66.2	268	184	68.7	0.479
Respiratory symptoms medication	523	168	32.1	268	119	44.4	0.001
Vitamins	523	122	23.3	268	4	1.5	0.000
Analgesic medication	523	65	12.4	268	13	4.9	0.001
Gastrointestinal symptom medication	523	17	3.3	268	0	0.0	0.003
Asthma medication	523	15	2.9	268	8	3.0	0.926
Diabetes medication	523	3	0.6	268	0	0.0	0.288

* GIT; Gastrointestinal tract.

## Data Availability

The data presented in this study are available on request from the corresponding author. The data are not publicly available due to restrictions on privacy.

## References

[1] Yezli S., Yassin Y.M., Awam A.H., Attar A.A., Al-Jahdali E.A., Alotaibi B.M. (2017). Umrah. An Opportunity for Mass Gatherings Health Research. Saudi Med. J..

[2] Ahmed Q.A., Arabi Y.M., Memish Z.A. (2006). Health Risks at the Hajj. Lancet.

[3] Yezli S., Assiri A.M., Alhakeem R.F., Turkistani A.M., Alotaibi B. (2016). Meningococcal Disease during the Hajj and Umrah Mass Gatherings. Int. J. Infect. Dis..

[4] Yezli S., Zumla A., Yassin Y., Al-Shangiti A.M., Mohamed G., Turkistani A.M., Alotaibi B. (2017). Undiagnosed Active Pulmonary Tuberculosis among Pilgrims during the 2015 Hajj Mass Gathering: A Prospective Cross-Sectional Study. Am. J. Trop. Med. Hyg..

[5] Memish Z.A., Zumla A., Alhakeem R.F., Assiri A., Turkestani A., al Harby K.D., Alyemni M., Dhafar K., Gautret P., Barbeschi M. (2014). Hajj: Infectious Disease Surveillance and Control. Lancet.

[6] Shujaa A., Alhamid S. (2015). Health Response to Hajj Mass Gathering from Emergency Perspective, Narrative Review. Turk. J. Emerg. Med..

[7] Ezat S., Puteh W., al Salem A., Radwan N.A., Dahliah S., Zannah A. (2019). The Causes of Malaysian and Indonesian Pilgrims’ Hospitalization during the Hajj Season 1440 (2019) in Mena and Arafat Hospitals. Saudi J. Biomed. Res. Abbreviated Key Title Saudi J. Biomed. Res..

[8] Khan I.D., Khan S.A., Asima B., Hussaini S.B., Zakiuddin M., Faisal F.A. (2018). Morbidity and Mortality amongst Indian Hajj Pilgrims: A 3-Year Experience of Indian Hajj Medical Mission in Mass-Gathering Medicine. J. Infect. Public Health.

[9] Yezli S., Elganainy A., Awam A. (2018). Strengthening Health Security at the Hajj Mass Gatherings: A Harmonised Hajj Health Information System. J. Travel Med..

[10] Statistic South Africa Community Survey 2016 IN BRIEF. http://www.statssa.gov.za/wp-content/uploads/2016/03/CS-FW-ID.pdf.

[11] Alzeer A.H. (2009). Respiratory Tract Infection during Hajj. Ann. Thorac. Med..

[12] Aleeban M., Mackey T.K. (2016). Global Health and Visa Policy Reform to Address Dangers of Hajj during Summer Seasons. Front. Public Health.

[13] Pane M., Yin Mei Kong F., Bayu Purnama T., Glass K., Imari S., Samaan G., Oshitani H. (2004). Indonesian Hajj Cohorts and Mortality in Saudi Arabia From. J. Epidemiol. Glob. Health.

[14] Al-Ghamdi S.M.G., Akbar H.O., Qari Y. (2003). Pattern of Admission to Hospitals during Muslim Pilgrimage (Hajj) Sleep Disorders View Project Liver Fibrosis View Project. Saudi Med. J..

[15] Khan N.A., Ishag A.M., Ahmad M.S., El-Sayed F.M., Bachal Z.A., Abbas T.G. (2006). Pattern of Medical Diseases and Determinants of Prognosis of Hospitalization during 2005 Muslim Pilgrimage (Hajj) in a Tertiary Care Hospital. A Prospective Cohort Study. Saudi Med. J..

[16] Al Shimemeri A. (2012). Cardiovascular Disease in Hajj Pilgrims. J. Saudi Heart Assoc..

[17] Yezli S., Alotaibi B.M., Saeed A.A.B. (2016). The Hajj Health Requirements: Time for a Serious Review?. Lancet.

[18] Sridhar S., Benkouiten S., Belhouchat K., Drali T., Memish Z.A., Parola P., Brouqui P., Gautret P. (2015). Foot Ailments during Hajj: A Short Report. J. Epidemiol. Glob. Health.

[19] Kim H.-Y. (2017). Statistical Notes for Clinical Researchers: Chi-Squared Test and Fisher’s Exact Test. Restor. Dent. Endod..

[20] Ebrahim S.H., Memish Z.A., Uyeki T.M., Khoja T.A.M., Marano N., McNabb S.J.N. (2009). Pandemic H1N1 and the 2009 Hajj. Science.

[21] General Authority for Statistics (Saudi Arabia) Hajj Statistics 2017. https://www.stats.gov.sa/sites/default/files/haj_1438.pdf.

[22] Verhoeven P.O., Gautret P., Haddar C.H., Benkouiten S., Gagnaire J., Belhouchat K., Grattard F., Charrel R., Pozzetto B., Drali T. (2015). Molecular Dynamics of Staphylococcus Aureus Nasal Carriage in Hajj Pilgrims. Clinical Microbiology and Infection.

[23] Zhang Y., Shi F., Yu Z., Yang A., Zeng M., Wang J., Yin H., Zhang B., Ma X. (2019). A Cross-Sectional Study on Factors Associated with Hypertension and Genetic Polymorphisms of Renin-Angiotensin-Aldosterone System in Chinese Hui Pilgrims to Hajj. BMC Public Health.

[24] Gautret P., Benkouiten S., Griffiths K., Sridhar S. (2015). The Inevitable Hajj Cough: Surveillance Data in French Pilgrims, 2012–2014. Travel Med. Infect. Dis..

[25] Parker S., Parker S. (2010). The Hajj: A Constant Travel Destination amidst Changing Times. South. Afr. J. Epidemiol. Infect..

[26] Yezli S., Mushi A., Yassin Y., Maashi F., Khan A. (2019). Knowledge, Attitude and Practice of Pilgrims Regarding Heat-Related Illnesses during the 2017 Hajj Mass Gathering. Int. J. Environ. Res. Public Health.

[27] Pane M., Imari S., Alwi Q., Nyoman Kandun I., Cook A.R., Samaan G. (2013). Causes of Mortality for Indonesian Hajj Pilgrims: Comparison between Routine Death Certificate and Verbal Autopsy Findings. PLoS ONE.

[28] Alqahtani A.S., Althimiri N.A., BinDhim N.F. (2019). Saudi Hajj Pilgrims’ Preparation and Uptake of Health Preventive Measures during Hajj 2017. J. Infect. Public Health.

[29] UNU-WIDER Morné Oosthuizen UNU-WIDER: Research Brief: Racial Inequality and Demographic Change in South Africa. https://www.wider.unu.edu/publication/racial-inequality-and-demographic-change-south-africa.

[30] Yezli S., Mushi A., Almuzaini Y., Balkhi B., Yassin Y., Khan A. (2021). Prevalence of Diabetes and Hypertension among Hajj Pilgrims: A Systematic Review. Int. J. Environ. Res. Public Health.

[31] Hoang V.T., Ali-Salem S., Belhouchat K., Meftah M., Sow D., Dao T.L., Ly T.D.A., Drali T., Ninove L., Yezli S. (2019). Respiratory Tract Infections among French Hajj Pilgrims from 2014 to 2017. Sci. Rep..

[32] Tashani M., Barasheed O., Azeem M., Alfelali M., Badahdah A.-M., Bokhary H., Almasri N., Alshehri J., Matbouly G., Kalantan N. (2014). Pneumococcal Vaccine Uptake Among Australian Hajj Pilgrims in 2011-13. Infect. Disord. Drug Targets.

[33] Razavi S.M., Sabouri-Kashani A., Ziaee-Ardakani H., Tabatabaei A., Karbakhsh M., Sadeghipour H., Mortazavi-Tabatabaei S.A., Salamati P. (2013). Trend of Diseases among Iranian Pilgrims during Five Consecutive Years Based on a Syndromic Surveillance System in Hajj. Med J. Islamic Repub. Iran.

[34] (2015). World Health Organization GHO|by Category|Raised Blood Pressure (SBP ≥ 140 OR DBP ≥ 90), Age-Standardized (%)—Estimates by Country [Internet]. https://www.who.int/diabetes/country-profiles/zaf_en.pdf?ua=1.

[35] Madani T.A., Ghabrah T.M., Albarrak A.M., Alhazmi M.A., Alazraqi T.A., Althaqafi A.O., Ishaq A.H. (2007). Causes of Admission to Intensive Care Units in the Hajj Period of the Islamic Year 1424 (2004). Ann. Saudi Med..

[36] Yezli S., bin Saeed A.A., Assiri A.M., Alhakeem R.F., Yunus M.A., Turkistani A.M., Booy R., Alotaibi B.M. (2016). Prevention of Meningococcal Disease during the Hajj and Umrah Mass Gatherings: Past and Current Measures and Future Prospects. Int. J. Infect. Dis..

[37] Saudi Arabia Ministry of Health 1441H.-Hajj Season—Health Regulations. https://www.moh.gov.sa/en/Hajj/Pages/HealthRegulations.aspx.

[38] Benkouiten S., Al-Tawfiq J.A., Memish Z.A., Albarrak A., Gautret P. (2019). Clinical Respiratory Infections and Pneumonia during the Hajj Pilgrimage: A Systematic Review. Travel Med. Infect. Dis..

[39] Hoang V.-T., Dao T.-L., Duc Anh Ly T., Belhouchat K., Larbi Chaht K., Gaudart J., Mmadi Mrenda B., Drali T., Yezli S., Alotaibi B. (2019). The Dynamics and Interactions of Respiratory Pathogen Carriage among French Pilgrims during the 2018 Hajj. Taylor Francis.

[40] Lessler J., Reich N.G., Brookmeyer R., Perl T.M., Nelson K.E., Cummings D.A. (2009). Incubation Periods of Acute Respiratory Viral Infections: A Systematic Review. Lancet Infect. Dis..

[41] Salmon-Rousseau A., Piednoir E., Cattoir V., de La Blanchardière A. (2016). Infections Liées Au Hadj. Médecine et Maladies Infectieuses.

[42] Centers for Disease Control and Prevention Food Poisoning Symptoms|Food Safety|CDC. https://www.cdc.gov/foodsafety/symptoms.html.

[43] Yezli S., Zaraa S., Yassin Y., Mushi A., Stergachis A., Khan A. (2020). Medication Utilization Pattern among Outpatients during the Hajj Mass Gathering. Saudi Pharm. J..

[44] Hoang V.T., Nguyen T.T.T., Belhouchat K., Meftah M., Sow D., Benkouiten S., Dao T.L., Anh Ly T.D., Drali T., Yezli S. (2019). Antibiotic Use for Respiratory Infections among Hajj Pilgrims: A Cohort Survey and Review of the Literature. Travel Med. Infect. Dis..

[45] Hoang V.T., Meftah M., Anh Ly T.D., Drali T., Yezli S., Alotaibi B., Raoult D., Parola P., Pommier de Santi V., Gautret P. (2019). Bacterial Respiratory Carriage in French Hajj Pilgrims and the Effect of Pneumococcal Vaccine and Other Individual Preventive Measures: A Prospective Cohort Survey. Travel Med. Infect. Dis..

[46] Yezli S., Khan A., Bouchama A. (2019). Summer Hajj Pilgrimage in the Era of Global Warming: A Call for Vigilance and Better Understanding of the Risks. J. Travel Med..

[47] Alqahtani A.S., Wiley K.E., Tashani M., Willaby H.W., Heywood A.E., BinDhim N.F., Booy R., Rashid H. (2016). Exploring Barriers to and Facilitators of Preventive Measures against Infectious Diseases among Australian Hajj Pilgrims: Cross-Sectional Studies before and after Hajj. Int. J. Infect. Dis..

[48] Badahdah A.-M., Alghabban F., Falemban W., Albishri A., Rani Banik G., Alhawassi T., Abuelizz H., Bakarman M.A., Khatami A., Booy R. (2019). Meningococcal Vaccine for Hajj Pilgrims: Compliance, Predictors, and Barriers. Trop. Med. Infect. Dis..

[49] Bieh K.L., Khan A., Yezli S., El-Ganainy A., Asiri S., Alotaibi B., Ghallab S., Elkholy A., Abubakar A., Jokhdar H. (2020). Implementing the Health Early Warning System Based on Syndromic and Event-Based Surveillance at the 2019 Hajj. East. Mediterr. Health J..

[50] Alotaibi B.M., Yezli S., bin Saeed A.A.A., Turkestani A., Alawam A.H., Bieh K.L. (2017). Strengthening Health Security at the Hajj Mass Gatherings: Characteristics of the Infectious Diseases Surveillance Systems Operational during the 2015 Hajj. J. Travel Med..

[51] Soon S., Svavarsdottir H., Downey C., Jayne D.G. (2020). Wearable Devices for Remote Vital Signs Monitoring in the Outpatient Setting: An Overview of the Field. BMJ Innov..

[52] Zhao F., Li M., Tsien J.Z. (2015). Technology Platforms for Remote Monitoring of Vital Signs in the New Era of Telemedicine. Expert Rev. Med Devices.

[53] Alghamdi S., Alqahtani J., Aldhahir A. (2020). Current Status of Telehealth in Saudi Arabia during COVID-19. J. Fam. Community Med..

[54] Jokhdar H., Khan A., Asiri S., Motair W., Assiri A., Alabdulaali M. (2020). COVID-19 M Itigation Plans D Uring H Ajj 2020: A S Uccess S Tory of Z Ero C Ases. Health Secur..

